# The effects of physical activity interventions on preschoolers’ self-regulation skills: a systematic review and meta-analysis of randomized controlled trials

**DOI:** 10.1186/s12966-026-01960-7

**Published:** 2026-08-05

**Authors:** Maureen Su-Ling Lim, Ng Ee Lynn, Yiling Song, Kylie D. Hesketh, Wei-Peng Teo

**Affiliations:** 1 Physical Education and Sport Science Academic Department, National Institute of Education, Nanyang Technological University, 1 Nanyang Walk, Singapore, 637616 Singapore; 2 Centre for Research in Child Development (CRCD), National Institute of Education, Nanyang Technological University, Singapore, Singapore; 3 Department of Physical Education, Tsinghua University, Beijing, China; 4 Institute for Physical Activity and Nutrition, School of Exercise and Nutrition Sciences, Deakin University, Geelong, Australia; 5 Science of Learning in Education Centre (SoLEC), National Institute of Education, Nanyang Technological University, Singapore, Singapore

**Keywords:** Physical activities, Cognitive regulation, Emotional regulation, Behavioural regulation, Intensity, MVPA, Physical activity parameters

## Abstract

**Background:**

Previous studies have shown that physical activity (PA) benefits self-regulation (SR) in preschoolers. However, few studies have examined the effects of PA parameters such as intensity, duration, and delivery modality on domains of SR such as emotional, behavioural and cognitive SR. This study aimed to synthesize evidence from randomized controlled trials (RCT) to evaluate the effects of PA-interventions on SR-domains, and to identify intervention parameters most effective for SR development in preschool-aged children.

**Methods:**

Systematic literature search was conducted from February through to November 2025 across Web of Science, PsycINFO, and ERIC databases. Eligible studies included RCTs involving PA-interventions with preschoolers aged 18 to 71.99 months that assessed SR-domains. Of 2,510 studies screened and 192 full-text articles reviewed, 25 RCTs met inclusion criteria and were included in the meta-analysis. 24 studies were school-based interventions, and one was home-based. Data extraction, risk of bias, and quality assessments were independently conducted by two reviewers using the Cochrane RoB-2 (RoB-2) tool and QualSyst.

**Results:**

Overall, meta-analyses showed that PA-interventions produced a small to moderate improvement in SR (*g* = 0.549, *p* < 0.01), with positive significant effects for behavioural SR (*g* = 0.452, *p* < 0.01), cognitive SR (*g* = 0.428, *p* < 0.01), and multi-SR-domains (*g* = 1.039, *p* < 0.01). Emotional SR was not significantly impacted (*g* = 0.390, *p* = 0.148). Subgroup analyses showed that both low-intensity (*g* = 0.620, *p* < 0.001) and moderate-to-vigorous PA (*g* = 0.388, *p* < 0.01), and group-based PA-interventions (*g* = 0.502, *p* < 0.01) significantly improved SR, but individual PA-interventions showed non-significant effects (*g* = 0.218, *p* = 0.189). Publication bias was indicated by Egger’s regression (*t* = 3.892; *p* < 0.01) and 21 studies had “*some concerns*,” three were “*high*” risk, and one “*low*” risk indicated by RoB-2, while QualSyst rated 15 studies as “*strong*” and 10 as “*good*.”

**Conclusions:**

PA-interventions significantly enhance SR in preschoolers, particularly when more than one SR-domain was examined. There were moderate benefits for cognitive and behavioural SR but limited effectiveness for emotional SR. Both low-intensity PA and MVPA are effective, and group-based interventions may be more impactful than individual delivery.

**Supplementary Information:**

The online version contains supplementary material available at https://doi.org/10.1186/s12966-026-01960-7.

## Background

### Self-regulation

Self-regulation (SR) is a multifaceted skill that emerges in early childhood and underpins young children's learning and development across cognitive, emotional, and behavioural domains. Cognitive SR involves processes such as attention control and goal-directed problem-solving; emotional SR encompasses modulation of affective responses; and behavioural SR includes inhibition of impulsive actions and task persistence. These domains draw on overlapping neural systems, particularly prefrontal cortex networks, which support higher-order control of thoughts, emotions, and behaviour [1–3]. In everyday context, SR enables children to regulate and adapt their attention, emotions and behaviour to situational demands, for example, sustaining focus during classroom task, waiting their turn, or resolving conflicts constructively during peer play [1, 2, 4]. The coordination of cognitive, emotional, and behavioural regulation supports more complex, integrated forms of control that are foundational for learning, social relationships, and school readiness [5–7].

### Physical activities (PA) and SR

Early PA helps physical growth and plays a critical role in supporting cognitive, social, and emotional development [8, 9], contributing to school readiness and success later in life [10–13]. Current evidence strongly supports the role of PA in the development of SR skills, however, the relationships between PA parameters such as intensity, type, duration and delivery modality and SR-domains remain underexplored. When children aged three to five engage in moderate-to-vigorous PA (MVPA) intensity, they exhibit stronger SR skills and better school readiness [10, 12]. Specifically, when routine structured PA type is integrated into the classroom curriculum or practiced at home, children have shown improvement in attention, task engagement, executive functions (EF), social skills, emotional and behavioural regulation [8, 9].

The duration and intensity of PA children participate in is an important factor associated with the development of their SR skills. A recent systematic review examining young children from various countries who met the PA guidelines recommended by Canada, Australia, and the World Health Organization (toddlers: at least 180 min of physical activity daily; children aged three and older: at least 180 min including one-hour of MVPA) demonstrated better emotional and behavior regulation, cognitive abilities, and social skills [14]. Furthermore, cross-sectional studies that examine PA-intensity, type and duration found that preschool children who engaged in longer duration of structured MVPA showed stronger associations with SR development [10, 15, 16]. Conversely, children who do not engage frequently in MVPA or do not meet the PA guidelines were found to have poorer motor skills, increased risks of health issues, poorer EF, SR and social skills [14, 17]. In the absence of sufficient environmental stimulation, including limited engagement in PA, developmental trajectories may be compromised. This can result in maladaptive brain functioning [18] and delays in SR development [19]. Such delays can negatively impact learning, social-emotional competencies, and school readiness [20–22].

The conceptual framework proposed by Lubans et al. (2016) suggests that SR is an important pathway linking PA to cognitive and psychosocial outcomes. Within this framework, PA is hypothesized to enhance SR by engaging neurobiological systems that support executive control and stress regulation, which in turn influence psychosocial functioning and cognition [23]. Thus, examining SR as an outcome variable is theoretically grounded, as it captures the behavioural expressions of underlying neurocognitive improvements elicited by PA.

### Aim of this systematic review and meta-analysis

Previous systematic reviews and meta-analyses have consistently demonstrated that PA-interventions improve SR and EF in young children. However, several notable gaps remain. Existing empirical studies and reviews primarily centred on the cognitive dimensions of SR, frequently framing it as a subset of EF and emphasizing capacities such as working memory, inhibitory control, and cognitive flexibility, leaving behavioural and emotional aspects of SR underexplored [24–28]. For example, both Wei et al. [24] and Mao et al. [25] focused their meta-analyses on the cognitive aspects of SR, specifically EF such as working memory, inhibitory control, and cognitive flexibility. Their work highlights the positive effects of cognitively engaging PA-interventions on these cognitive functions in children. However, their study largely equates SR with EF, overlooking the critical behavioural and emotional domains that contribute to a more complete understanding of SR [24, 25]. Furthermore, there was notable variability in how PA intervention parameters were defined across the two domains. Wei et al. [24] omitted delivery modality, Mao et al. [25] omitted intensity, and some included studies lacked control groups, resulting in inconsistent reporting and limited clarity on optimal intervention characteristics. Building on these insights, the present systematic review and meta-analysis extend the scope by examining PA effects across all SR-domains and by adopting a standardized framework to extract and report PA parameters consistently across studies, thereby addressing this gap in prior research. Therefore, this systematic review and meta-analysis aim to address these gaps:

1. How do PA parameters influence SR outcomes in preschool aged children?
2. Which specific domain of SR, including emotional, behavioural, or cognitive, is most effectively enhanced by PA-interventions in preschool aged children?

By addressing current inconsistencies in intervention designs and reporting, our findings will facilitate more precise recommendations for future research and practice.

## Methods

### Search strategies

A systematic search of published literature was conducted across three databases (Web of Science, PsycINFO, and ERIC) as specified in the registered protocol. No date restrictions were applied to any database, ensuring that records from database inception were included. Preliminary searches were conducted between February and June 2025, with the final comprehensive search updated through the pre-registered end date of 30 November 2025, prior to manuscript submission. In total, 3,056 studies were identified and imported into Covidence, where 546 duplicates were removed, yielding 2,510 studies for screening by two independent reviewers (ML and SYL). This systematic review was registered with the Open Science Framework (OSF; https://doi.org/10.17605/OSF.IO/3NJVX) and adhered to the Preferred Reporting Items for Systematic Review and Meta-Analyses (PRISMA 2020) guidelines. Table 1 summarises the keywords used for the search strings. The Boolean operators “NOT” and “NOT ALL” and exclusion terms such as “Autism,” “ASD,” “Autism Spectrum Disorder,” “Attention Deficit Hyperactivity Disorder,” “Hyperactivity,” “ADHD,” “Adolescence,” and “Elderly” were employed to remove irrelevant studies (see Appendix for complete search strings). Broad PA terms (e.g., physical activit*, exercise*, play*) were selected in line with established preschool PA reviews to encompass sport-based activities without redundancy [29]. For each included study, intensity (low vs high) was coded according to the classification used in the original study, which typically reflected the prescribed type and/or dose of physical activity (e.g., light/moderate vs vigorous activity, lower vs higher weekly frequency or session duration, or author-defined low- vs high-dose programmes). A single physiological cut-off was not imposed across studies; instead, each study’s own operationalisation of intensity was retained to preserve consistency with the original intervention design.

**Table 1:** Summary of key terms used in each database

Physical activity keywords	Self-regulation keywords	Age group keywords
“Physical activity” “Exercise” “Active play” “Play-based activity” “Movement-based activity”	“Self-regulation” “Behavioural regulation” “Emotional regulation” “Cognitive regulation” “Self-control” “Executive function” “Inhibitory control” “Task-switching” “Switching” “Updating” “Shifting” “Set shifting” “Cognitive flexibility” “Working memory” “Effortful control”	“Preschool” “Young children” “Early childhood” “Child” “Kindergarten” “Day care” “Child care”

### Inclusion and exclusion criteria

There were no restrictions on the publication years of the included studies and a total of seven studies [30–36] were published between 2000–2019, and 18 studies [37–54] were published from 2020 onwards.

This review applied the following criteria for included studies:

1. Healthy, neurotypical children aged 18–71.99 months (up to 6 years).
2. Children attending kindergarten, preschool, or equivalent early childhood education setting.
3. Experimental designs: RCTs, cluster RCTs, or pilot RCTs.
4. Studies with quantitative measurement of PA parameter and its association with SR.
5. Studies that assessed at least one domain of SR (cognitive, emotional, or behavioural), or executive function (EF).
6. Written in or translated into English.
7. Peer-reviewed and published in academic journals.

The exclusion criteria for the review were as follows:

1. Children diagnosed with medical, developmental, or neurodiverse conditions.
2. Non-peer-reviewed sources: conference abstracts, dissertations, commentaries, books, clinical advice, animal or methodological studies.
3. Studies with non-quantitative PA parameters or SR outcomes.
4. Observational, protocol-only, cross-over designs and studies with no control.
5. Non-RCTs studies.
6. Age criteria not met at any measurement point.
7. Children who have started primary/elementary school.

### Data selection process

**Fig. 1:**
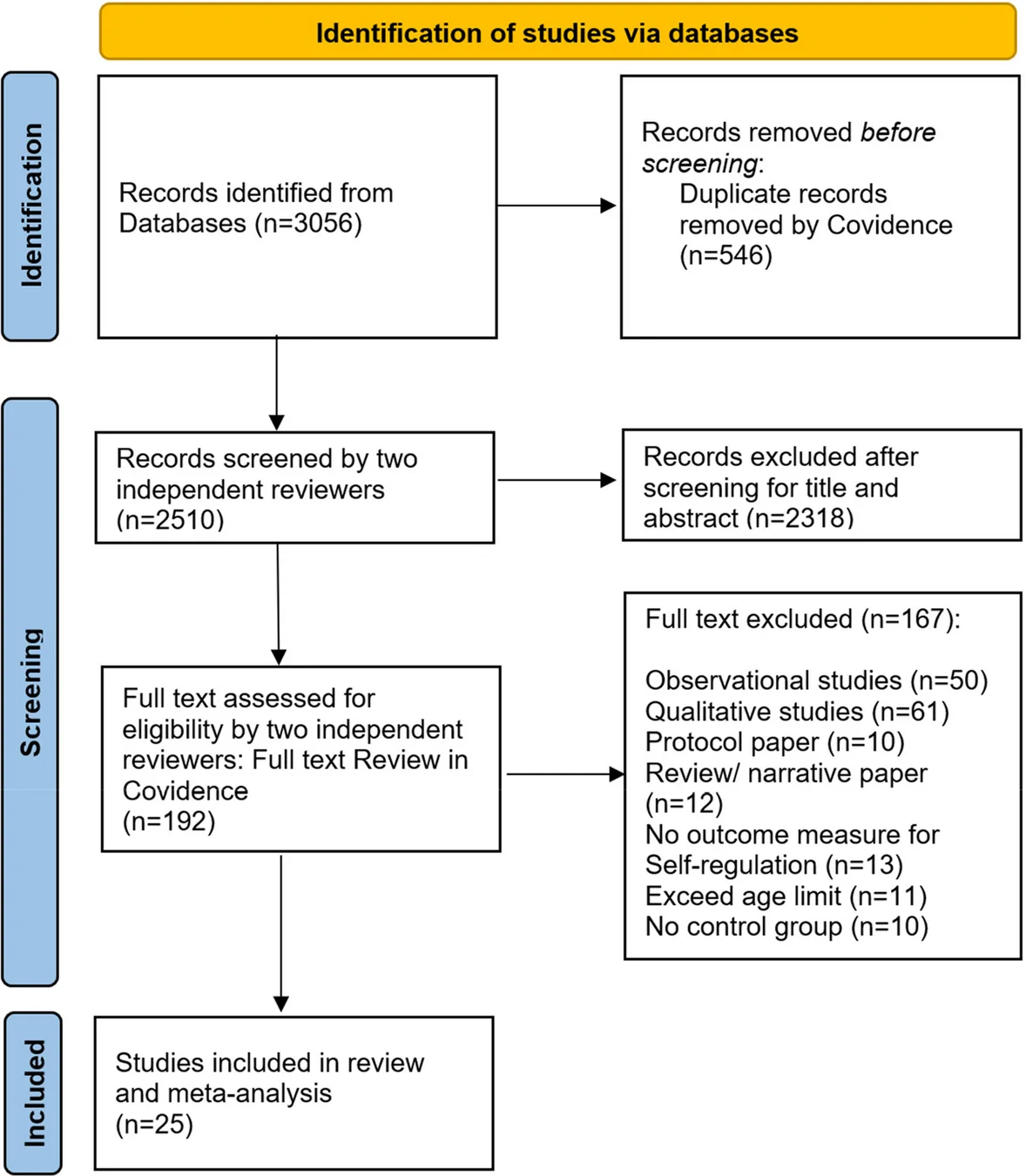
Flow chart of the systematic review process adhering to PRISMA

Figure 1 (PRISMA flow diagram) illustrates the search process and Supplementary Table 1 represents the search strings of keywords to narrow the search to attain the studies. Two reviewers (ML and SYL) independently screened the titles and abstracts and excluded studies that did not meet the eligibility criteria using Covidence. Subsequently, both reviewers independently assessed the full text of the remaining studies, and pilot trials were identified as studies explicitly labelled as 'pilot,' 'feasibility,' or 'exploratory’. Reviewers ensured that there were no duplicates or overlaps of studies in the included studies by cross-checking first/last authors, publication years, trial registration numbers, and follow-up publications. Any discrepancies were first discussed between the two reviewers, and if consensus could not be reached, a third reviewer (WPT) was consulted to resolve the disagreement.

### Definitions

For this study, PA parameters were defined as: duration (time/session), frequency (number of sessions completed) [24, 27, 55], and delivery modality (group or one-on-one settings). Intensity was classified as light/low (slow walking, stretching) [42, 56] or MVPA/high (relay races, running, and chasing games) [42, 56] was coded according to the original authors' classification, regardless of measurement method (accelerometer, observation, or descriptive), with accompanying details noted where available (e.g., target heart rate, heart rate intensity, activity examples). No post-hoc physiological cut-offs were imposed across studies to preserve each intervention's original operationalization [42, 56]. PA type was classified along two orthogonal dimensions: (1) structure (structured vs unstructured) and (2) cognitive engagement (cognitively engaging vs non-cognitively engaging). This yielded four combinations: structured–cognitively engaging, structured–non-cognitively engaging, unstructured–cognitively engaging, and unstructured–non-cognitively engaging. Of 25 included studies, 24 were both structured and cognitively engaging. PA type referred to structured, unstructured, cognitively engaging and non-cognitively engaging. This approach reduced variability in PA definitions and clarified how PA parameters impacted SR development [1]. SR-domains were classified as cognitive, emotional, behavioural and multi-domain. The multi-domain category included studies that used SR assessment tools measuring more than one SR-domain.

### Data extraction process

The following information was standardized and extracted using Microsoft Excel: (1) author’s name and year of publication; (2) Sample characteristics: Sample size and age or age range of participants; (3) PA-intervention parameters: type, frequency, intensity, delivery modality and measurement instruments; (4) SR assessment: focus (attention shifting, working memory, cognitive flexibility, inhibition, emotional/behavioural regulation) and measurement instrument (5) Study outcomes: Overall effectiveness of the intervention.

### Study quality appraisal: QualSyst quality assessment and risk of bias (RoB-2)

This study used two complementary tools to assess study quality: the QualSyst checklist, which evaluates general methodological rigor across quantitative designs including RCTs, and the Cochrane Risk of Bias 2 (RoB2) tool, which assesses bias specifically in RCTs. QualSyst complements RoB2 by addressing challenges unique to preschool interventions, where RoB2’s strict criteria often make low risk ratings difficult in nonclinical behavioural studies [57–59]. The QualSyst checklist includes 14 items scored as *Yes-(2)*, *Partial-(1)*, or *No-(0)*, excluding Not Applicable items. Scores are calculated as a proportion of the maximum and categorized as *strong (*> *0.80)*, *good (0.71–0.79)*, *adequate (0.50–0.70)*, or *limited (*< *0.50)* [58, 60]. The RoB2 tool evaluates five bias domains using signalling questions, rating studies as *low risk*, *some concerns*, or *high risk* of bias. Risk of bias was assessed using RoB-2 for all trials, and domain-level ratings for pilot versus non‑pilot RCTs. Discrepancies were resolved through discussion as described earlier and a third reviewer (WPT) was consulted if needed.

### Statistical analyses procedures

Meta-analysis was implemented using Comprehensive Meta-Analysis (version 3.0) [61]. Hedges’ *g* with 95% confidence interval (CI) was computed for each study. As some intervention groups in the included studies had fewer than 20 participants [32, 35, 41, 43], Hedges’ *g* was used instead of Cohen’s *d* [62]. Hedges’ *g* [63] and random effects model were performed on all the included studies assessing intervention effects. Separate subgroup random effects SR-domains (Cognitive [C], Behavioural [B], Emotional [E], and Multi), delivery modality (Group or Individual) and PA-intensity (Low or MVPA). Forest plots were generated separately to assess the effect of the PA-interventions on these moderators. A fourth subgroup analysis examined whether the role of the intervention deliverer (teacher, trained instructor, or researcher) moderated PA-intervention effects on SR. As several studies reported more than one SR-domain outcome, each contributing a separate effect size under the same intervention leader, the total number of effect sizes entered into this analysis (k = 35) exceeded the total number of included studies (k = 25). One effect size using a co-led format (teacher and trained instructor) was reported descriptively but excluded from the formal between-group comparison due to insufficient power (k = 1). Age and gender were also considered as candidate moderators of the PA–SR relationship but were not subjected to formal moderator analysis. Age was excluded due to minimal variability across the included studies, as samples were restricted to the preschool period (approximately 3–6 years) and predominantly reported as a single mean rather than a distribution or range, providing insufficient variance for a meaningful meta-regression. Gender was excluded because only two of the 25 included studies [37, 40] reported PA effects on SR disaggregated by sex; the remaining 23 studies reported pooled, mixed-sex outcomes only, precluding a statistically reliable subgroup or meta-regression analysis.

Accounting for the random-effects model used, heterogeneity of effect sizes was assessed using *Q* statistics and *p*-values. Two meta-regression analyses were performed between (a) duration (in minutes) and the *g* statistics of PA-interventions, and (b) number of sessions and the *g* statistics. Potential publication bias was assessed via a funnel plot and Egger’s regression test, whereby no bias is indicated by a non-significant asymmetrical graph. Duval and Tweedie’s trim and fit method was used to impute the missing studies and estimate the predicted effect size for asymmetric funnel plot outcomes. An alpha level of *p* < 0.05 was used to determine significance. A sensitivity analysis was also conducted to examine the influence of pilot study inclusion on pooled effect estimates, both overall and within SR-domain subgroups (see Supplementary Table 2 for Sensitivity Analysis).

## Results

### Description of the included studies characteristics

#### Study characteristics

Twenty-five studies were included for this review based on the inclusion criteria and of these, all studies provided sufficient data to be incorporated into the meta-analyses, including seven explicitly labelled pilot/feasibility studies (total sample *n* > 50). A detailed summary of the studies included is provided in Table 2. All 25 studies involved children aged three years and older, with an overall mean age of 4.72 years. Four studies [32, 35, 41, 43] had less than 20 participants in the intervention group and 13 studies [30–32, 34, 35, 39, 41–43, 49, 50, 52, 53] had less than 40 participants in the intervention group. 24 studies were school-based interventions and one [32] study was home-based. Regarding intervention delivery, 20 studies [30, 33–35, 37–46, 48–52, 54] implemented group-based interventions, while five [31, 32, 36, 47, 53] were delivered individually. Among the included studies, information on the teacher-to-participant ratio was not reported in 12 studies [33–35, 39, 40, 44–46, 48, 51, 52]. As for who led the interventions, 10 studies [31, 37–39, 42, 43, 45, 50, 51, 54] were conducted by teachers, one study [34] was co-led by teachers and trained instructors, seven studies [32, 35, 36, 47, 49, 52, 53] were conducted by researchers and seven studies [30, 33, 40, 41, 44, 46, 48] were conducted by trained instructors.

**Table 2:** Sensitivity analysis comparing pooled effect sizes with and without pilot studies

Analysis	k	Hedges’g	95% CI	*p*-value
Primary (all studies)	25	0.549	[0.276, 0.821]	< 0.001
Restricted (pilots excluded)	18	0.811	[0.279, 1.342]	0.003

Included studies were categorised into the four SR-domains. 12 studies [30–32, 34, 35, 37, 42, 44, 46, 49, 52, 54] examined only the cognitive domain of SR, two studies [33, 43] examined only the behavioural domain of SR. There were no studies specifically examining emotional SR. A total of 10 studies examined multi-SR-domains [36, 38, 39, 41, 45, 47, 48, 50, 51, 53].

PA parameters include PA-intensity, duration (time spent per session), frequency (number of sessions completed), delivery modality (in group or individually) and PA type. 24 studies [30–36, 38–54] provided details for all the PA parameters except one study [37], which failed to provide duration per session and frequency of intervention. Six studies [33, 37, 39, 40, 51, 52], despite providing delivery modality, were unclear on the exact number of children per group when the study mentioned the intervention was delivered in groups. It is important to note that although data on both family and school delivery modality were collected, these categories were not analysed separately, as this distinction did not address the research questions of this study.

Seven studies [30, 37, 38, 41, 43, 45, 54] delivered low PA-intensity interventions and 21 studies [31–36, 39–44, 46–54] delivered MVPA-interventions. Of these, three studies [41, 43, 54] had both PA-intensity types as part of the intervention and hence the total number of studies exceeded 25. All 25 studies described delivery modality with 20 studies [30, 33–35, 37–46, 48–52, 54] conducted in group settings while five studies [31, 32, 36, 47, 53] delivered the intervention individually.

PA and SR measures from the included studies were categorized into two groups: direct objective measures, where children were assessed using standardized tests, and indirect subjective measures, where parents or teachers reported on the children’s performance in SR and/or PA. Four studies [37, 38, 48, 51] implemented both direct (e.g. DCCS, Stroop, Go/No-Go, HTKS) and indirect (e.g. teacher/parent report-BRIEF-P, SSRS, SDQ) measurement of SR, 19 studies [30–36, 39–44, 46, 47, 50, 52–54] implemented only direct measures and two studies [45, 49] implemented only indirect measures. For measurement of PA, eight studies [37, 40, 41, 43, 48–50, 53] did not include any PA outcome measures, 13 studies [31–34, 36, 39, 42, 44, 46, 47, 51, 52, 54] implemented direct measures (e.g. accelerometers, TGMD, MABC-2), and four studies [30, 35, 38, 45] implemented indirect measures (e.g. Early Years Movement Skills Checklist, PMSC) only. It is noteworthy that this review did not analyze PA type and intervention setting (home- versus school-based) as 24 of the 25 identified studies were cognitively engaging, structured and school-based. These limitations resulted in insufficient variability for meaningful subgroup analysis and would not have addressed the research questions. The descriptive characteristics of included studies can be found in Table 3.

**Table 3:** Descriptive characteristics of included studies

#	Studies	RCT or Pilot RCT	N intervention	N Control	Mean Age (in years)	PA assessment	Intensity	Frequency	Duration		Total sessions (Mins)	Delivery modality	Intervention led by	SR assessment tool	SR-domain assessed	Intervention outcome
1	Kats Gold et al., 2021 [37]	Pilot RCT	49	43	Intervention: 5.53 Control: 5.36	Nil	Low	Daily (Nil)	28 weeks		Nil	Group (School) Group size not stated	Teachers	Hearts and Flowers	C	Effective
2	Xiong et al., 2019 [30]	Pilot RCT	30	30	Intervention: 4.65 Control: 4.38	PSPCSA*	Low	5X/week (20 min)	8 weeks		40 (160 min)	Group (School) 2–4 per group	Trained instructors	Dimensional change card sort	C	Effective
3	Wen et al., 2018 [31]	RCT	29	28	Intervention: 4.35 Control: 4.47	Accelerometer: Actigraph GT1M	MVPA	5X/week (20 min)	10 weeks		50 (1000 min)	Individual (School)	Teachers	Go/No Go Spatial conflict arrow (SCA) Working memory span task (WMS) Flexible item selection (FIS)	C C C C	Not Effective
4	Vazou & Mavilidi, 2021 [38]	Pilot RCT	138	135	Intervention: 4.31 Control: 4.13	PMSC*	Low	5X/week (10.6 min)	8 weeks		40 (426 min)	Group (School) 16–18 per group	Teachers	Social skills rating scale (SSRS) questionnaire Stroop like- Day Night task	E and B (multi-SR-domain) C	Partial
5	Zhang et al., 2024 [39]	RCT	31	32	Intervention: 5.52 Control: 5.57	Accelerometer: Polar FT2	Moderate	3X/week (60 min)	12 weeks		36 (2160 min)	Group (School) Group size not stated	Teachers	Go/No Go Snack delay task Wrapped gift task Stop signal task Circle drawing task Dots task Wrapped gift task Snack delay task	B B C C C C E E	Effective
6	Gao et al., 2019 [32]	RCT	18	14	Intervention: 4.56 Control: 4.93	Accelerometer: Actigraph GT3X +	MVPA	5X/week (30 min)	12 weeks		60 (1800 min)	Individual (Home)	Researchers	Dimensional change card sort	C	Effective
7	Robinson et al., 2016 [33]	RCT	68	45	Intervention: 4.52 Control: 4.30	TGMD-2	MVPA	3X/week (40 min)	5 weeks		15 (600 min)	Group (School) Group size not stated	Trained instructors	Delay Snack task	B	Effective
8	Hirata 2023 [40]	RCT	1256	554	Intervention: 5.07 Control: 5.07	Nil	MVPA	3X/week (30–40 min)	20 weeks		60 (2100 min)	Group (School) Group size not stated	Trained instructors	Attention by cancelling test (ACT) Working memory test for children (WMTC) Trails test	C C C	Effective
9	Fisher et al., 2011 [34]	Pilot RCT	34	30	Intervention: 6.1 Control: 6.2	Accelerometer: Actigraph GT1M	MVPA	2X/week (30 min)	10 weeks		20 (1200 min)	Group (School) Group size not stated	Teachers and Trained instructors	Cambridge neuropsychological test battery (CANTAB) Attention network test (ANT) Cognitive assessment system (CAS)	C C C	Effective
10	Jarraya et al., 2022 [41]	RCT	PMR: 18 PE-:17	17	Intervention group 1 (PMR): 5.40 Intervention group 2 (PE): 5.40 Control: 5.40	Nil	PMR- Low PE- MVPA	2X/week (30 min)	12 weeks		24 (720 min)	Group (School) 18 per group	Trained instructors	Statue test Teddy Bear Cancellation Test Rey Simple Figure Test	B C C	PMR: Effective PE: Not Effective
11	Bai et al., 2022 [42]	RCT	30	32	Intervention: 4.16 Control: 4.70	Motor skills	MVPA	3X/week (50 min)	8 weeks		24 (1200 min)	Group (School) 8 per group	Teachers	Silly sound stroop (SSS) task Empty house task Dimensional change card sort	C C C	Effective
12	Ureña et al., 2020 [43]	RCT	Locomotion with Bicycle (BL): 12 Locomotion Without Bicycle (WBL): 13 Dramatic Story group (DS): 12	12	Intervention group 1 (BL): 4.49 Intervention group 2 (WBL): 4.55 Intervention group 3 (DS): 4.63 Control: 4.47	Nil	BL-MVPA WBL-moderate DS- low	1 session (15 min)	Nil		1 (15 min)	Group (School) 12–13 per group	Teachers	HTKS*	B	Effective
13	Zhang et al., 2022 [44]	RCT	57	52	Intervention: 4.51 Control: 4.53	MABC-2*	Moderate	3X/week (40 min)	12 weeks		36 (1440 min)	Group (School) Group size not stated	Trained instructors	Modified 1-back task	*C*	Effective
14	Alesi et al., 2021 [45]	RCT	110	64	Intervention: 5.23 Control: 5.18	Early Years Movement Skills Checklist	Low	3X/week (60 min)	10 weeks		30 (1800 min)	Group (School) Group size not stated	Teachers	IDPA- Questionnaire for the Early Identification of Learning Difficulties	All domain (multi-SR-domain)	Effective
15	Olive et al., 2024 [46]	RCT	170	144	Intervention: 4.27 Control: 4.31	Accelerometers- ActiGraph GT3X +	MVPA	5X/week (15 min)	22 weeks		110 (2310 min)	Group (School) Group size not stated	Trained instructors	Go/No Go Mr. Cucumber task Dimensional change card sort	C C C	Effective
16	Xiong et al., 2017 [35]	RCT	19	20	Intervention: 4.67 Control: 4.67	PSPCSA*	MVPA	5X/week (30 min)	12 weeks		60 (1800 min)	Group (School) Group size not stated	Researchers	Preschool Executive Function Measure	C	Effective
17	Haas et al., 2022 [47]	Pilot RCT	51	52	Intervention: 5.76 Control: 5.66	Electrocardiograph device: ekgMove	MVPA	1 session (20 min)	1 session		1 (20 min)	Individual (School)	Researchers	HTKS* Day-night Stroop-like task	B C	Not Effective
18	Veiga et al., 2023 [48]	RCT	153	78	Intervention: 5.07 Control: 5.08	Nil	MVPA	2X/week (40 min)	5 weeks		20 (800 min)	Group (School) Group size not stated	Trained instructors	Day and Night task HTKS* Strengths and Difficulties Questionnaire (SDQ)	C C B	Effective
19	Pewdee et al., 2024 [49]	Pilot RCT	36	36	Intervention: 4.44 Control: 4.58	Nil	MVPA	2X/week (20–30 min)	9 weeks		18 (445 min)	Group (School) 6 per group	Researchers	Mahidol University EF-101	C	Effective
20	Liu et al., 2022 [50]	RCT	24	24	Intervention: 4.92 Control: 4.88	Nil	Moderate	5X/week (30 min)	4 weeks		20 (600 min)	Group (School) 6 per group	Teachers	Mr. Ant Go/No Go Card sorting task	C B C	Effective
21	Mavilidi et al., 2023 [51]	RCT	CPA: 55 COG: 48	41	Intervention group 1 (CPA): 4.34 Intervention group 2 (COG): 4.44 Control: 4.41	Accelerometer: ActiGraph GT3X +	MVPA	2X/week (17–20.5 min)		6 weeks	12 (225 min)	Group (School) Group size not stated	Teachers	Mr Ant Go/No Go Strengths and Difficulties Questionnaire (SDQ)	C C B	Not Effective
22	Wang et al., 2022 [52]	RCT	30	30	Overall: 4–5 years old Did not provide information for intervention and control	CNPFSM*	Moderate	4X/week (40 min)		12 weeks	48 (1920 min)	Group (School) Group size not stated	Not stated	Panda/Lion test Grass-Snow task Corsi Blocks Test Reverse Corsi Blocks Test Flexible Item Selection Task (FIST)	C C C C C	Effective
23	Giordano & Alesi, 2022 [53]	Pilot RCT	Cognitively-engaging PA: 25	Control 1- (free play, non-cognitively engaging physical activity): 25 Control group 2 (no additional physical activity beyond regular school activities): 25	Intervention: 5.68 Control: 5.68	Nil	Moderate	3X/week (15 min)		6 weeks	18 (270 min)	Individual (School)	Researchers	Day-Night Stroop test HTKS* Gift wrap task Snack delay task	C B and C (multi-SR-domain) E E	Effective
24	Stein et al., 2017 [36]	RCT	48	53	Intervention: 6.02 Control: 6.03	MABC-2* Heart rate monitor: Polar RS800sd	Moderate	1 session (25 min)		1 session	1 (25 min)	Individual (School)	Researchers	Hearts and Flowers Simon says	C B	Not Effective
25	Schmidt et al., 2020 [54]	RCT	Physical-cognitive group (combined physical and cognitive training): 75 Intervention- Cognitive group (sedentary cognitive training):52	62	Intervention group 1 (Physical-cognitive): 5.39 Intervention group 2(Cognitive) mean age: 5.35 Control: 5.28	MABC-2 Accelerometer: movisens	Combined Physical-Cognitive Training- Moderate Sedentary Cognitive Training- Low	4X/week (15 min)		6 weeks	24 (360 min)	Group (School) 13–14 per group	Teachers	Pictorial n-back task Stroop-like day-night task Dimensional change card sort	C C C	Partial: Effective for Updating; Not effective for Inhibition and Shifting

^*^Abbreviations: *PSPCSA* Pictorial Scale of Perceived Competence and Social Acceptance for Young Children, *PMSC* The pictorial scale of Perceived Movement Skill Competence, *TGMD-2* Test of gross motor development-Second edition, *MABC-2* Movement assessment battery for children- Second edition, *CNPFSM* China’s National Physical fitness standard manual, *HTKS* Head-Toes-Knees-Shoulders task, *B* BehaviouralSR, *C* Cognitve SR, *E* Emotional SR

### Meta-analysis of the included studies

#### Meta-analysis of effects of PA-interventions on overall SR and individual SR-domains

**Fig. 2:**
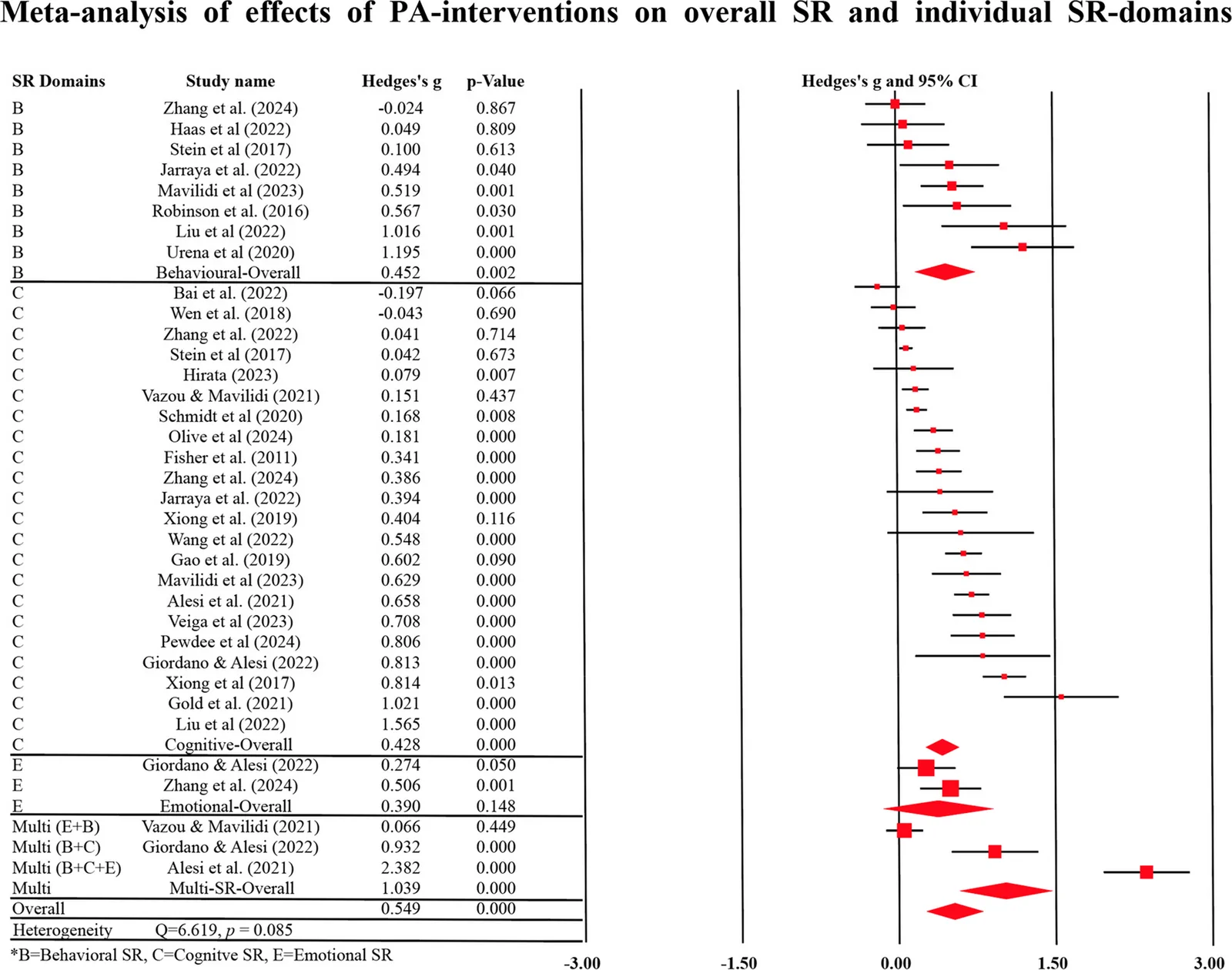
Forest plot for meta-analysis on the overall effect of PA-intervention on SR-domains

Figure 2 shows the overall effects of PA-interventions on SR. The meta-analyses revealed a positive moderate and statistically significant PA-intervention effect on overall SR-domains (*g* = 0.549, 95% CI[0.276–0.821], *p* < 0.01). The SR-domains were categorized into four distinct groups: Cognitive, Behavioural, Emotional and Multi. Eight studies examined the effect of PA-intervention on the behavioural domain of SR and there was a significant moderate effect (*g* = 0.452, 95% CI[0.165–0.740], *p* = 0.002). 22 studies examined the cognitive domain of SR and there was a significant moderate effect (*g* = 0.428, 95% CI[0.268–0.589], *p* < 0.01). Two studies examined emotional domain of SR, revealing a small-to-moderate effect (*g* = 0.390), and this effect was not statistically significant (95% CI[−0.139–0.918], *p* = 0.148). Three studies examined more than one domain of SR (multi-SR-domain) and there was a significant large effect (*g* = 1.039, 95% CI[0.594–1.484], *p* < 0.01). Of these 3 studies [38, 45, 53], one study [45] examined all three domains (C, B and E) of SR, one study [38] examined E and B domains and one study [53] examined B and C domains of SR. The results from the random-effects model were used and it indicated that the heterogeneity among the three SR-domains was not significant (*Q* = 6.619, *p* = 0.085) and there was no difference in intervention effects among the four SR-domains.

### Sensitivity analysis

To examine the influence of pilot study inclusion on the pooled effect estimate, a sensitivity analysis was conducted comparing the primary meta-analysis (k = 25, including 7 pilot studies, g = 0.549, 95% CI [0.276, 0.821], *p* < 0.001) against a restricted analysis excluding the 7 pilot studies (k = 18, full RCTs only; g = 0.811, 95% CI [0.279, 1.342], *p* = 0.003). This restricted analysis substantially altered subgroup composition: the emotional domain subgroup was reduced from 2 to 1 study and the multi-domain subgroup from 3 to 1 study, while the behavioural (8 to 7) and cognitive (22 to 16) domain subgroups remained adequately powered for pooling. The marked increase in point estimate and substantially wider confidence interval in the restricted analysis reflect this loss of precision in the emotional and multi-domain subgroups, rather than a true difference in the underlying PA–SR relationship. A one-study-removed sensitivity analysis across all 25 studies further supported this interpretation: pooled effect sizes remained stable (g = 0.514–0.535) when six of the seven pilot studies were individually removed, with the largest single deviation occurring when Vazou and Mavilidi (2021) (*n* = 273), was removed (g = 0.655). This study, the largest of the seven pilot trials and larger than 16 of the 18 full-scale RCTs included in this review, contributed to both the multi-domain and cognitive subgroups; its disproportionate influence on the multi-domain subgroup (one of only three contributing studies) explains its outsized effect on the restricted analysis above. Collectively, these results suggest that the primary pooled estimate (k = 25) is robust to pilot study inclusion at the individual-study level, but that domain-specific estimates for emotional and multi-domain SR should be interpreted cautiously, particularly when pilot data are excluded.

### Effective PA-interventions and the PA parameters—PA-intensity

**Fig. 3:**
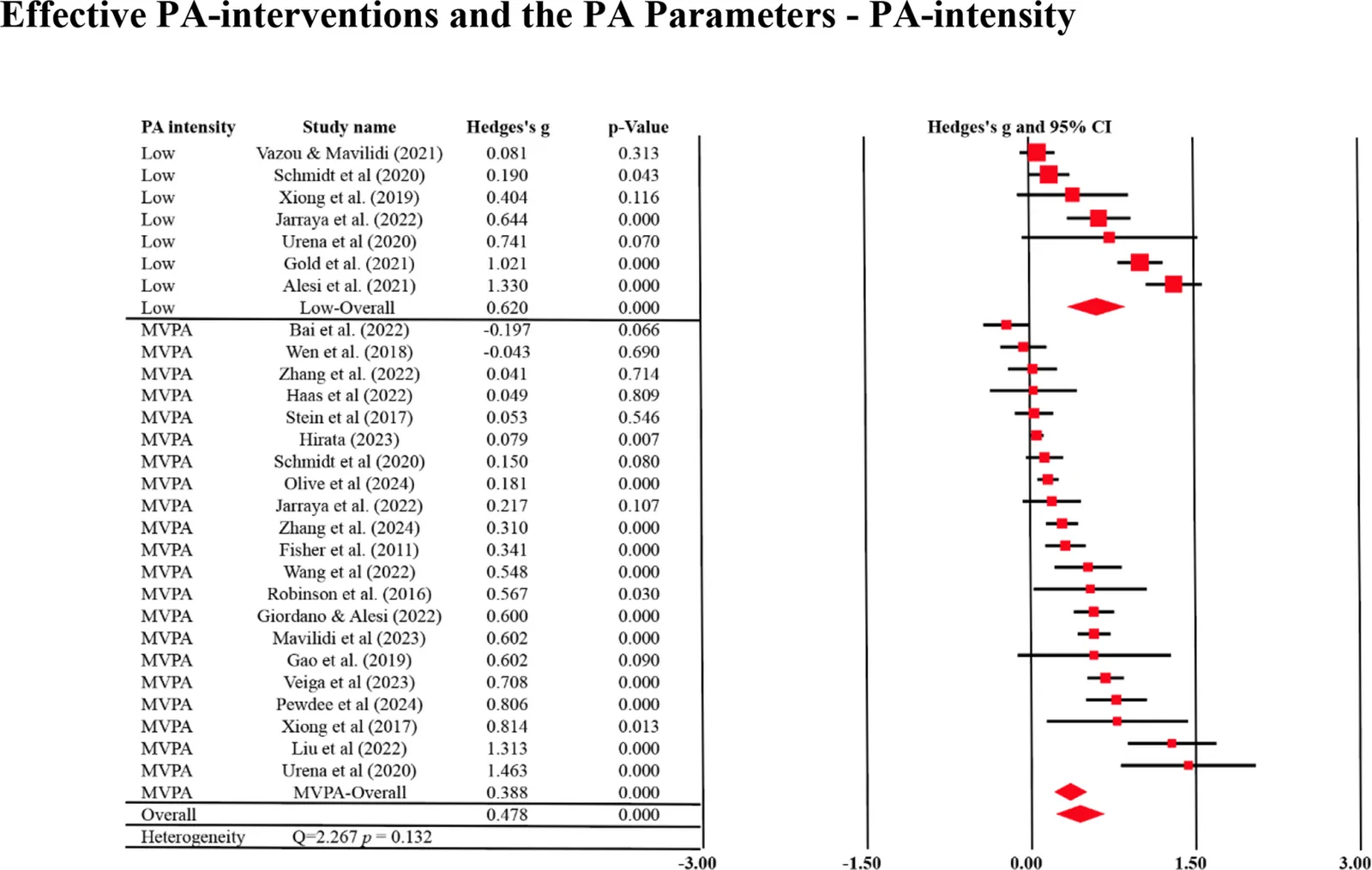
Forest plot for meta-analysis on the overall effect of PA-intervention on PA-intensity

Figure 3 shows that PA-intervention had a moderate and statistically significant overall effect regardless of the intensity (*g* = 0.478, 95% CI[0.256–0.699], *p* < 0.001). PA-intensities were categorised into two groups, Low and MVPA and it is important to note that some studies had more than one intervention group with different PA-intensities which accounts for the total number of studies exceeding 25. Across seven low intensity intervention studies, there was a significant moderate effect (*g* = 0.620, 95% CI[0.357–0.884], *p* < 0.001). In 21 MVPA-intensity intervention studies, there was a significant small effect (*g* = 0.388, 95% CI[0.240–0.536], *p* < 0.001). The heterogeneity in effect sizes between the two PA-intensity groups was not significant (*Q* = 2.267, *p* = 0.132), and hence there was no difference in intervention effects between the two PA-intensity groups.

### Effective PA-interventions and the PA parameters—delivery modality

**Fig. 4:**
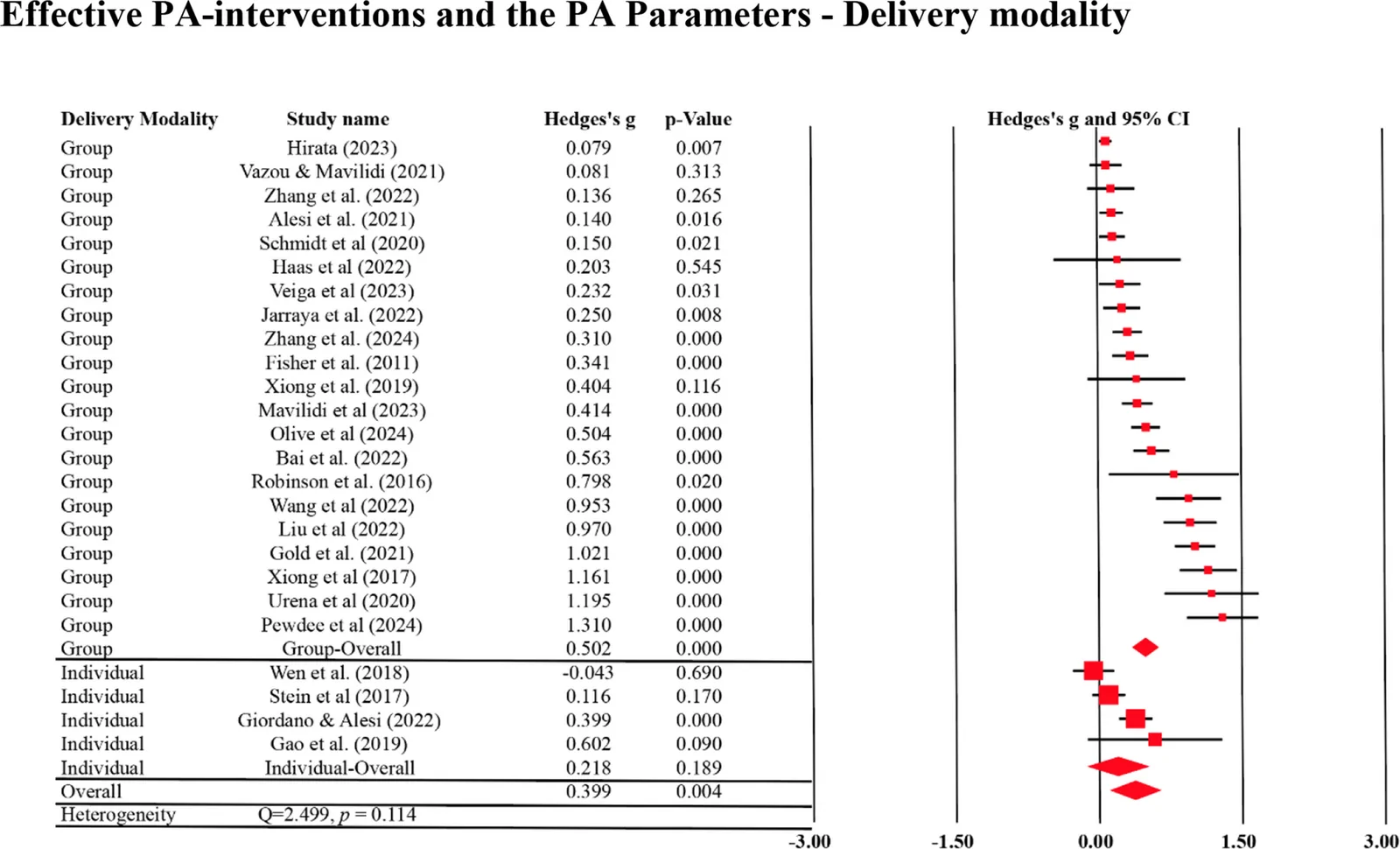
Forest plot for meta-analysis on the overall effect of PA-intervention by delivery modality

Figure 4 shows that PA-intervention had a small and statistically significant overall effect regardless of delivery modality in groups or individually (*g* = 0.399, 95% CI[0.131–0.667], *p* = 0.004). There was a small and non-significant effect (*g* = 0.218, 95% CI[−0.107–0.542], *p* = 0.189) for four studies using individually delivered interventions. In comparison, a moderate and statistically significant effect (*g* = 0.502, 95% CI[0.363–0.641], *p* < 0.001) was found in 21 studies using group delivered interventions. Heterogeneity in effect sizes between the two delivery modalities was non-significant (*Q* = 2.499,* p* = 0.114), indicating no difference in effects across the two delivery modalities.

### Effective PA-interventions and the PA parameters—intervention leader

**Fig. 5:**
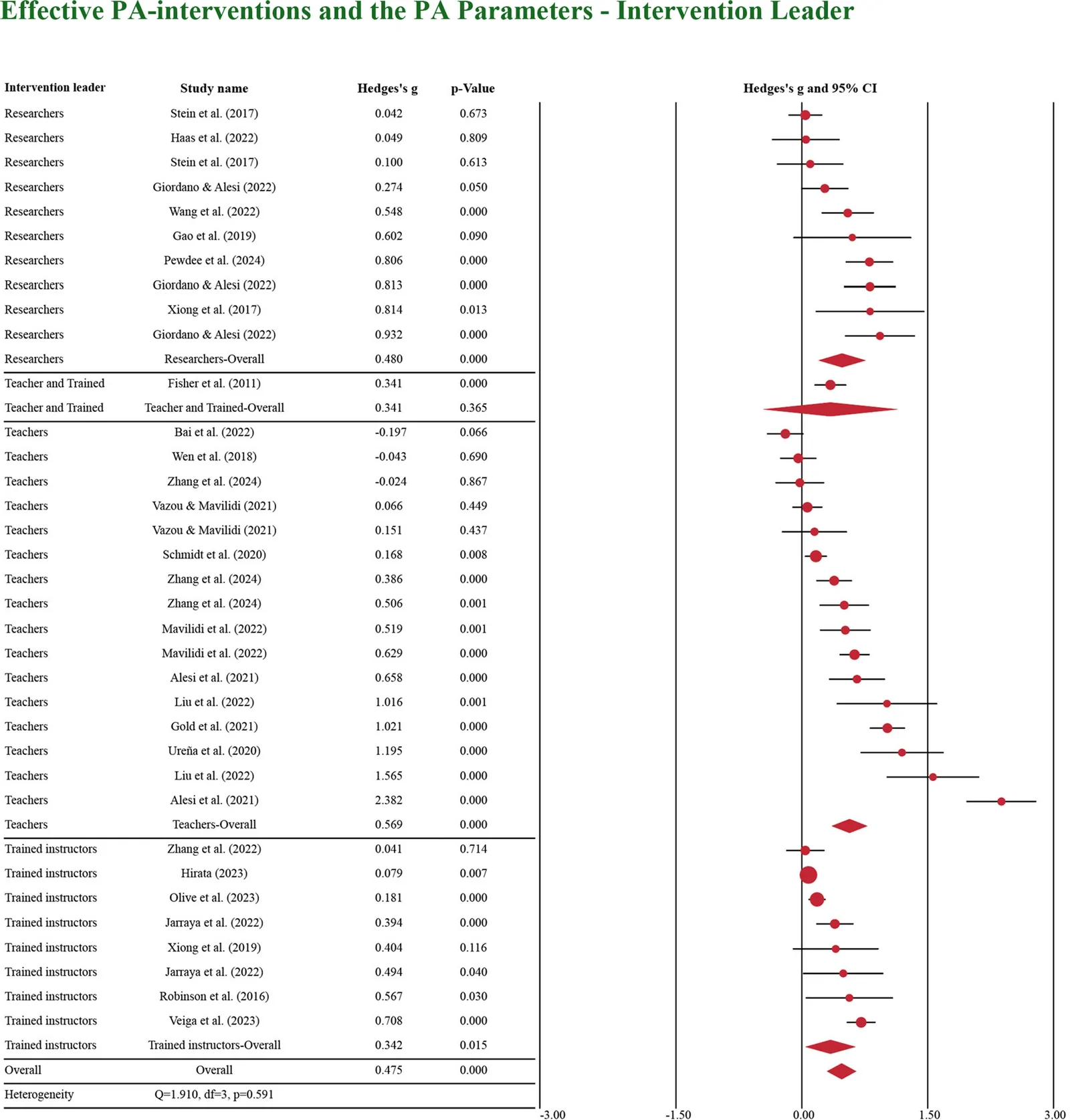
Forest plot for PA-intervention effects by intervention leader

Figure 5 shows the forest plot for PA-intervention effects by intervention leader. It is important to note that, as several studies reported more than one SR-domain outcome, the total number of effect sizes (k = 35) exceeded the total number of included studies (k = 25). PA-interventions led by teachers produced a significant moderate effect on SR (k = 16, g = 0.569, 95% CI[0.374–0.764], *p* < 0.001). Interventions led by researchers produced a significant small-to-moderate effect (k = 10, g = 0.480, 95% CI[0.223–0.737], *p* < 0.001), and interventions led by trained instructors produced a significant small effect (k = 8, g = 0.342, 95% CI[0.067–0.616], *p* = 0.015). One effect size used a co-led format (teacher and trained instructor; g = 0.341, 95% CI[−0.397–1.078], *p* = 0.365) and was not statistically significant, reflecting its single-study basis. Heterogeneity between the three primary leader categories was not significant (Q = 1.910, df = 3, *p* = 0.591), indicating no difference in intervention effects across leader types.

### Meta-regression on PA parameters: total time in minutes, number of sessions and duration in weeks

The total number of minutes were computed by totalling the duration (in minutes) of intervention and multiplying by the frequency of intervention (number of sessions per week and for how many weeks/months). For studies [40, 49, 51] that have a time range, the average time was used for that analysis. One study [37] was omitted from this analysis as it did not specify the time for the intervention sessions. Intervention duration was categorized as short (< 10 weeks), medium (10–12 weeks), or long (> 12 weeks). Three meta-regressions were carried out: 1) total intervention time in minutes, 2) total number of sessions and 3) duration of intervention in weeks, with all regression analyses showing non-significant findings, *R*^*2*^ = 0.018, *p* = 0.446, *R*^*2*^ = 0.017, *p* = 0.467 and *R*^2^ = −0.039, *p* = 0.566, respectively. Intervention duration ranged from a single session to 22 weeks, with session numbers varying from one to 154. Individual sessions lasted 10 min to two hours, yielding total intervention doses from less than one hour to over 60 h.

### Study quality appraisal and publication bias

**Fig. 6:**
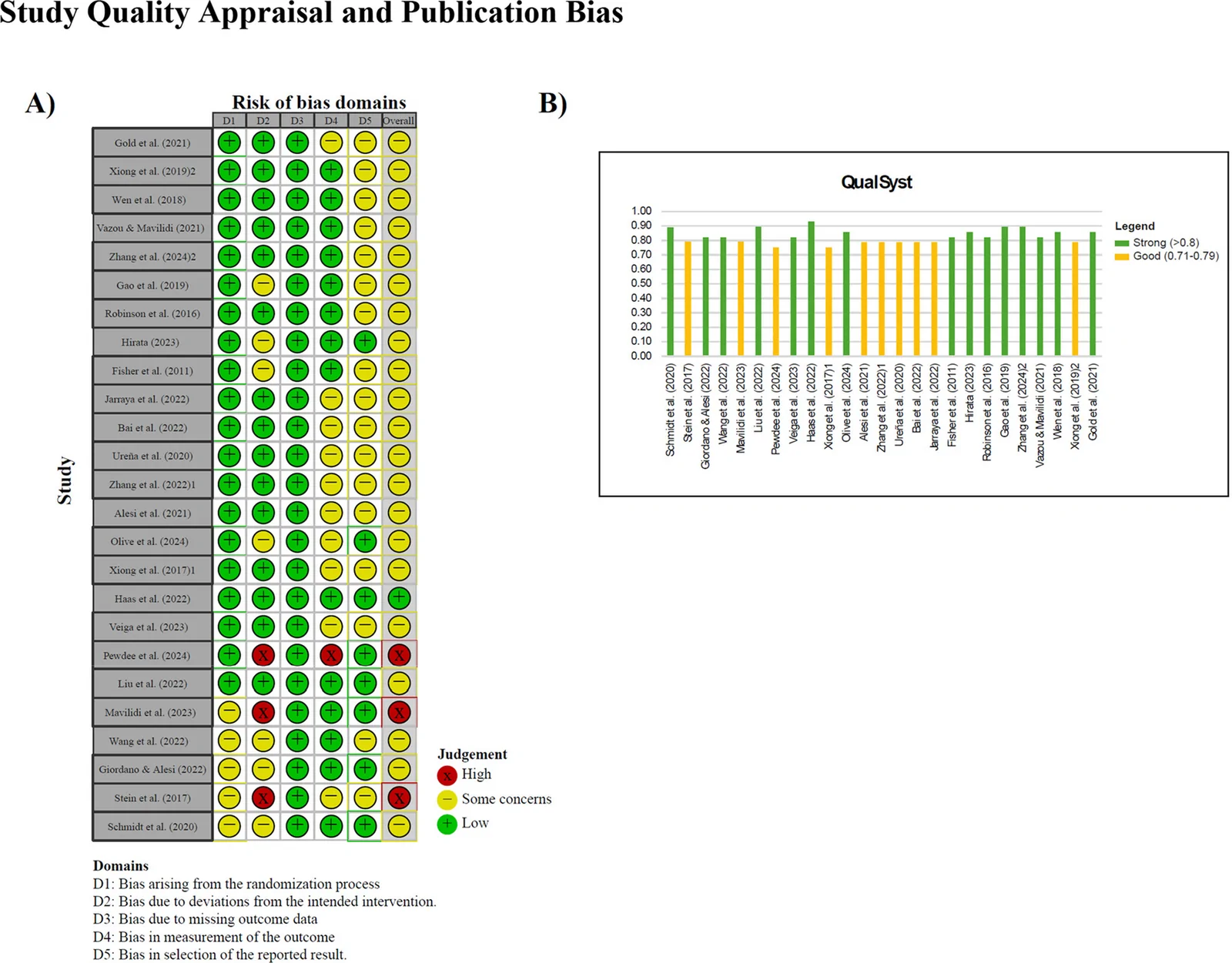
RoB-2 bias assessment and study quality QualSyst side by side comparison

**Fig. 7:**
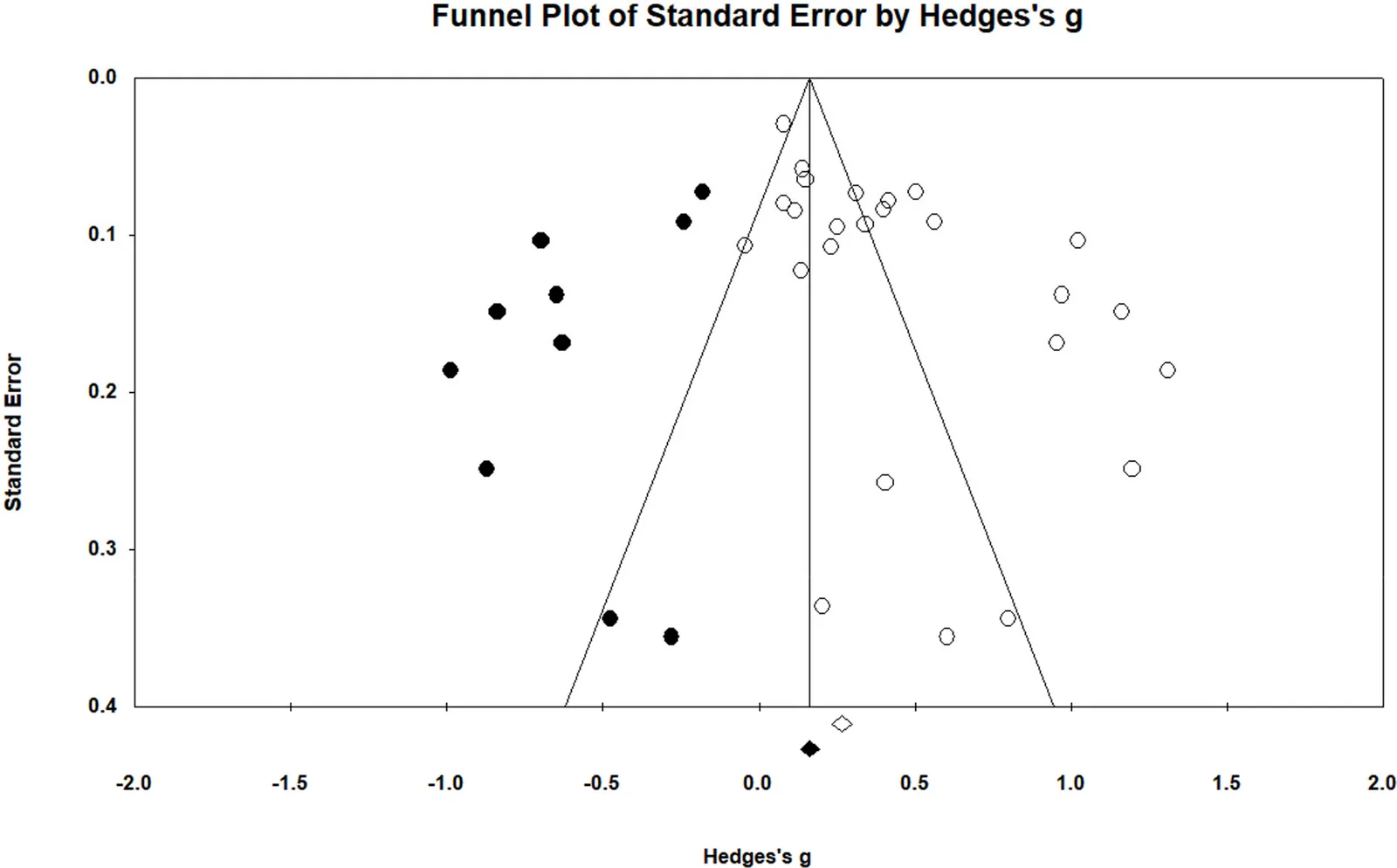
Funnel plot for publication bias with the random effects model. White dots represent included studies, and black dots represent imputed missing studies. The asymmetrical distribution indicates potential publication bias, with a greater proportion of positive results

Figure 6 shows a side-by-side comparison of the RoB-2 tool and QualSyst scores for the studies included. For the RoB-2 assessment, 21 studies were judged to have *“some concerns”* regarding risk of bias, three were rated as *“high”* risk, and only one was classified as *“low”* risk. It should be noted that the Cochrane RoB-2 tool is a stringent and rigorous assessment instrument, which often results in non-laboratory-based interventions being less likely to receive a “*low*” risk rating [57, 59]. The pattern of domain-specific RoB-2 ratings for the 7 pilot trials was broadly similar to that of the full RCTs, with most pilots rated low risk for randomization and missing outcome data, and predominantly ‘some concerns’ for deviations from intended interventions and selection of reported results. As for the QualSyst quality assessment, 15 papers were categorized as *“strong”* quality while 10 studies were categorized as *“good”* quality studies. Based on the funnel plot and Egger’s regression analysis in Fig. 7, the distribution of the included studies exhibited an asymmetrical trend indicating publication bias (*t* = 3.892; *p* = 0.001). Duval and Tweedie’s trim and fill analysis revealed that the adjusted effect size for the imputed studies was lower (*g* = 0.184, 95% CI[0.046–0.322]), as compared to the observed effect size (*g* = 0.457, 95%CI[0.332–0.582]).

## Discussion

The primary aim of this study was to identify the PA-intervention parameters most effective in enhancing overall SR outcomes in preschool-aged children. A secondary aim was to examine how these PA parameters influence distinct domains of SR, namely cognitive, emotional, and behavioural regulation among preschoolers. This meta-analysis highlights a novel contribution to the literature by demonstrating that specific PA-intervention parameters such as intensity and delivery modality were crucial in improving SR outcomes during early childhood. Overall, the results support earlier reviews showing beneficial effects of PA on SR related outcomes while extending them beyond cognitive domain.

The results of this review align with these previous conclusions, showing a consistent moderate positive effect of PA-interventions on the cognitive aspect of SR. However, the present study extends past work by focusing specifically on the influence of PA-intervention parameters on the various SR-domains rather than restricting analyses to the cognitive domain. For instance, while Cabral et al. [26] synthesized a larger number of studies [41], their focus was confined to cognitive and executive function components. Similarly, the reviews by Wei et al. [24] and Mao et al. [25], which included 24 and 23 studies respectively, were limited to cognitive SR. The current meta-analysis incorporated an additional study and uniquely examined the contribution of intervention parameters that were previously underreported or inconsistently reported across studies [24–28, 64, 65].

### PA parameters

This study examined the effects of key PA-intervention parameters, including intensity, delivery modality, and duration, on SR. Results showed a moderate effect of PA-intensity with small-moderate effects for MVPA and moderate effects for low intensity PA. Heterogeneity analyses found no significant differences between intensity group, indicating similar benefits across intensities. These findings align with past reviews reporting small to moderate effects for MVPA (D’Cruz et al. [66]; Cabral et al. [26]; Gordon et al. [65]), which considered a range of PA-intensities and types, not just MVPA. Notably, Li et al. [27] found that regular participation in PA had a small but meaningful effect on SR, suggesting that consistent engagement, rather than intensity alone, may be critical for SR development. Moreover, PA-intensity whether moderate to vigorous or light may be less relevant than maintaining children’s attention and engagement. Fisher et al. [67] found that young children often find it difficult to sustain MVPA and that light PA and MVPA independent variables may be quite similar in practice due to children’s overall low and fluctuating activity levels. Similarly, PA type classification used two orthogonal dimensions: 1) structural organization (structured vs unstructured) and 2) cognitive engagement (cognitively engaging vs non-cognitively engaging), yielding four categories based on each study's dominant descriptor. These dimensions are not mutually exclusive; teacher-led rule games were structured and cognitively engaging, while child-initiated play could be unstructured and cognitively engaging. Notably, 24/25 interventions were both structured and cognitively engaging, reflecting preschool norms. Therefore, interventions emphasizing regular, structured, cognitively engaging activities irrespective of intensity may better support SR development [67, 68].

Bishop et al. [69] recently proposed a standardized 5-category intensity framework (Very Low/Low/Moderate/High/Very High), which future studies should adopt for improved cross-study comparability [69]. However, retrospectively applying this framework was not feasible given the heterogeneous measurement methods (accelerometers, heart rate, self-reports) and pre-2025 terminology used across our included studies.

Building on the importance of examining different PA parameters, this review examined whether delivery modality influenced PA-intervention effects on overall SR. Although group-based delivery showed a moderate positive effect and individual delivery showed a non-significant effect, the between-group difference was not statistically significant. This suggests that, while group formats may appear more promising, the evidence does not support a definitive advantage over individually delivered interventions. Nevertheless, potential mechanisms underlying group-based benefits such as peer support, social interaction, cooperation, and shared accountability, remains theoretically relevant, as these factors can foster social-cognitive skills linked to SR [70]. Group interventions also promote attention, working memory, and emotional and behavioural regulation as children respond to social cues and peers, thus increasing cognitive engagement [42, 55]. Additionally, group-based formats may be more resource efficient and supportive for practitioners. These results should be interpreted cautiously due to the small number of individual delivery studies (*n* = 4) and potential confounders (i.e. children engaged in other concurrent PA or variations in teacher’s attitude towards PA) [66, 70, 71]. Future research comparing delivery formats is needed to confirm these findings. Building on this, whether the intervention deliverer moderated PA-intervention effects on SR was also examined. Teacher-led interventions produced the largest pooled effect, followed by researcher-led and trained-instructor-led interventions; however, these differences were not statistically significant (Q = 1.910, *p* = 0.591). This finding is encouraging for translational practice: teachers, who are already embedded in preschool settings, achieved comparable or greater effects than externally sourced trained instructors or researchers, suggesting that PA-SR interventions may not require specialist personnel to be effective. This has practical implications for scalability and cost, as classroom teachers represent a more sustainable and accessible delivery channel than research staff or contracted instructors. However, this interpretation should be tempered by the small number of effect sizes in the co-led format (k = 1) and potential confounding by training quality, fidelity of implementation, and teacher experience, which were not consistently reported across studies.

There was no significant influence of PA-intervention duration and total number of sessions on SR outcomes. Although non-significant, the data suggested a negative trend, indicating that more frequent sessions may diminish intervention effectiveness. This finding implies that simply increasing duration or frequency does not necessarily enhance SR development. The lack of significant results likely reflects the complex interplay of factors beyond duration and frequency. Consistent with Li et al. [27], our findings emphasize the importance of other variables such as activity intensity, cognitive engagement, and exercise structure in shaping outcomes. Overall, these results support a multidimensional perspective, suggesting thoughtfully structured activities, rather than simply longer or more frequent sessions, are most effective for promoting SR development.

### PA intervention on SR-domains

Our findings indicate that PA-interventions have a positive effect on SR in early childhood across cognitive, behavioural, emotional, and combined domains (multi). In contrast to previous meta-analyses, which primarily focused on the cognitive domain of SR or the cognitive aspects of EF, our study considered all major domains of SR. Prior reviews generally reported small- moderate positive effects (standardized mean difference or *g* approximately 0.30–0.35) exclusively for cognitive outcomes [25–28]. Consistent with earlier meta-analyses, our study found a comparable effect size for cognitive SR (*g* = 0.43), with the exception of the review by Wei et al., which found a larger estimate (g = 0.71) [24]. This consistency in effect sizes likely reflects the use of similar standardized tasks assessin*g* inhibition, working memory, and attention which are core components of cognitive SR. It is important to note, however, the considerable degree of overlap among primary studies included in previous meta-analyses [25–28], resulting in much of the evidence base being derived from a shared set of studies. This overlap may partly account for the observed similarity in estimates, meaning the apparent consistency in effect sizes likely reflects reliance on common datasets rather than truly independent replication of findings.

Notably, the larger effect size for cognitive SR reported by Wei et al. [24] may be due to stratification by narrower age groups (i.e., 3–4, 4–5, 5–6 years) and how intervention delivery modality was conceptualized and analysed. In their review, delivery method was defined as game-based versus non-game-based instruction, whereas in the present review, delivery modality was classified according to whether the intervention was implemented individually or in a group setting. These conceptual distinctions highlight the importance of clear and comprehensive reporting of intervention characteristics, as variations in definition can influence the identification and interpretation of moderating effects.

Past studies [24–28] often focus on cognitive SR, using assessment tools that capture both cognitive and behavioural aspects, reflecting overlapping constructs and measurement challenges. The reviewed studies employed diverse methods to measure SR, highlighting the lack of standardized outcome measures. Direct assessments involved objective tasks completed by children, such as the Flanker and Go/No-Go tasks, which could assess both behavioural SR and cognitive SR as these tasks also engages working memory, attention, and inhibitory control [72]. The effect size for behavioural(*g* = 0.452) and cognitive SR (*g* = 0.428) in our findings was similar and this further highlighted the issue of clearly distinguishing between SR subdomains, as overlapping task demands can blur conceptual boundaries and make categorization dependent on whether one follows the original study authors’ definitions or applies their own operational criteria. For instance, in the included studies, HTKS were employed by two studies [43, 48] as a measure of cognitive SR but it was taken as a measure of cognitive and behavioural SR in another study [53]. Similarly for the Go/No-Go task, four studies [31, 39, 46, 51] employed it as a measure of cognitive SR while one study [50] employed it as a measure of behavioural SR. Indirect measures relied on subjective reports from parents, teachers, or caregivers, using tools like the Behaviour Rating Inventory of Executive Function-Preschool Version(BRIEF-P) and Strengths and Difficulties Questionnaire (SDQ) to capture everyday regulatory behaviours which covers all three SR-domains. For instance, one study [45] in our review employed a questionnaire that examined cognitive, behavioural and emotional SR-domains. Theoretically, SR encompasses interconnected yet distinct domains. PA has been linked to direct influence on behavioural SR, as movement requires immediate regulation of impulses and responses, while cognitive SR benefits indirectly via enhanced executive functions like working memory and attention [11, 20, 73]. Developmentally, early childhood is a critical period of rapid EF maturation, making cognitive SR especially sensitive to PA-interventions [74, 75]. In contrast, indirect measures like caregiver reports capture broader, context-dependent regulatory behaviours influenced by adult scaffolding, adding subjective variance beyond objective tasks [66, 75]. Together, these theoretical and developmental insights explain differences in PA’s impact across SR-domains and measurement methods.

Our findings showed that the multi-SR-domain produced the largest effect size as compared to all the individual SR-domains, reinforcing the importance of examining SR holistically. However, this study acknowledges an important limitation that only four studies have assessed SR across multiple domains. Consequently, the effect size associated with the multi-SR-domain should be interpreted with caution, as it may represent an overestimation. It is important to acknowledge that behavioral, cognitive and emotional SR are closely intertwined, making it difficult to isolate and target a single domain [6, 76]. This overlap complicates research interpretation and limits practical application when targeting one domain exclusively [76]. Therefore, it is of paramount importance for future research to clearly define the SR-domain under investigation, specify the tools used, and consider examining all three domains (C, B, E) given the complex nature of SR.

Regarding emotional SR, the lack of a significant effect observed in our study may reflect the relatively small number of studies assessing this domain (*n* = 2). This highlights a broader gap in the literature, where behavioural and especially emotional SR are less frequently examined compared to cognitive SR, underscoring the need for future research to better explore and address these dimensions.

### Study quality appraisal

The RoB-2 assessment showed that most included RCTs (21 of 25) had *“some concerns”*, with three rated *“high risk”* and one rated *“low risk”*. This high number of *“some concerns”* reflects the challenges in controlling interventions delivered by teachers or professionals in school settings, and the stringent criteria of RoB-2 often yielded cautious ratings for nonclinical behavioural preschool interventions where blinding and strict protocol adherence are difficult to implement [57, 59]. QualSyst rated 15 studies as *“strong quality”* and 10 as *“good quality”*, indicating generally satisfactory but variable methodological rigor. Of the 25 RCTs, 7 (28%) were explicitly labelled pilot/feasibility studies in their primary publications, all with total sample sizes > 50 (range: 60–273). RoB-2 ratings for these pilot trials indicated low risk for randomisation in 6/7 and for missing outcome data in all 7, with mostly ‘some concerns’ in domains relating to deviations from intended interventions and selection of reported results. Overall, 5/7 pilot trials were judged as having ‘some concerns’, 1 as low risk and 1 as high risk, comparable to the pattern across the full set of RCTs. This substantially mitigates conventional underpowering concerns typically associated with pilots (*n* < 50 total). Visual inspection of forest plots confirmed no individual pilot exerted disproportionate influence on pooled effect estimates. A formal sensitivity analysis (see Results) substantiated this observation at the individual-study level (one-study-removed g range: 0.514–0.655) but revealed greater sensitivity at the subgroup level: excluding all 7 pilot studies increased the overall pooled effect to g = 0.811 (95% CI [0.279, 1.342]), driven primarily by the collapse of the emotional and multi-domain subgroups to a single study each. Notably, Vazou and Mavilidi (2021) (*n* = 273), was better powered than the majority of full-scale RCTs in this review, underscoring that pilot/RCT labelling does not necessarily reflect a study's statistical precision or evidentiary weight. Duplicate screening (cross-checking authors, years, registration numbers) identified no overlaps. A formal subgroup meta-analysis by pilot versus full RCT status was not conducted due to insufficient numbers of pilot trials (n = 7), which would have produced underpowered and imprecise estimates per standard meta-analytic guidelines. Given their small number (28% of total studies), moderate total sample sizes (> 50 per trial), and broadly comparable risk-of-bias profiles to full RCTs, the included pilot trials are unlikely to have materially distorted the pooled effects, although their preliminary status warrants cautious interpretation. Evidence of publication bias was apparent from funnel plot asymmetry and Egger’s regression test, and Duval and Tweedie’s trim and fill analysis indicated potential overestimation of the pooled effect size. These findings emphasize that the results should be interpreted within the confines of the methodological quality of the included studies. This variation may explain some of the differences seen across PA parameters and SR domains, though the overall positive PA-SR pattern remained consistent. Moreover, the presence of publication bias underlines the need for more rigorous, well-controlled studies and transparent reporting of null or negative findings.

### Limitations and future directions

One major limitation is the presence of publication bias in the included studies. The field shows a notable lack of published studies reporting null or non-significant results, which likely limited the available data and biased overall effect size estimates upward. This publication bias can lead to overestimated intervention effects, reducing the reliability and generalizability of conclusions.

A second key limitation is the study quality, with 21/25 RCTs rated as "some concerns" under RoB-2 and only one rated "low risk." Many studies faced typical challenges of preschool research, such as difficulty blinding assessors and ensuring consistent intervention delivery by teachers. This variation in study quality may help explain some of the mixed results we found across different PA parameters and SR domains. Although the overall pattern of results suggested positive PA–SR effects, these quality issues mean that the precise magnitude of the effects should be interpreted with caution.

Third, 24 of the 25 included studies were school-based interventions conducted mostly in formal preschool settings, restricting generalizability to other contexts such as home or community environments. Additionally, the small number of studies examining emotional and multi-SR-domains, as well as those employing individual delivery formats and studies with low PA-intensities, reduced the statistical power and further constrained the robustness of the subgroup analyses. Similarly, the co-led (teacher and trained instructor) subgroup for intervention leader was based on a single effect size, limiting interpretability of this category. This vulnerability was further confirmed by our sensitivity analysis, which showed that excluding pilot studies reduced the emotional and multi-domain subgroups to a single study each, and produced a markedly wider confidence interval for the overall pooled effect (95% CI [0.279, 1.342] vs. [0.276, 0.821]), highlighting that conclusions for these specific domains should be interpreted with particular caution. As detailed in the Statistical Analyses Procedures, age and gender could not be formally tested as moderators due to restricted age variability and limited sex-disaggregated reporting (only 2 of 25 studies) across included studies, leaving open whether PA–SR effects differ systematically between boys and girls or across narrower age bands within the preschool period. Finally, the interpretation of PA intensity was constrained by heterogeneous operational definition measurement approaches across studies (e.g., accelerometery, observation, author descriptions), which may have introduced additional misclassification bias and obscured potential dose–response patterns. Future research should ensure detailed and standardized reporting of PA parameters, including intensity, intervention design, duration, frequency, delivery modality, and demographic subgroup effects (gender, age), and clearly specify which types of SR assessments are used to evaluate each SR-domain and aim to examine all domains collectively rather than in isolation, given the closely intertwined nature of SR.

## Conclusion

This meta-analysis uniquely contributes to literature by comprehensively examining PA-intervention parameters and evaluating all domains of SR, rather than focusing on the cognitive aspects as in previous reviews conducted in 2024 [24, 25] and 2025 [26]. Our findings indicate that PA-interventions exert a moderate positive effect on preschoolers’ overall SR. The strongest effects were observed when multiple SR-domains were examined followed by behavioural and cognitive SR, where both yielded a moderate positive effect. There were inconclusive findings for emotional SR due to limited evidence. Effects were comparable across PA-intensities, suggesting that maintaining PA engagement may be more critical than intensity alone. Although group-based interventions appeared more effective than individual formats, caution is warranted as between-group analyses revealed no significant differences between these modalities. Together, these findings highlight the value of PA as a promising approach to enhance SR in early childhood, while emphasizing the need for more rigorous and transparent studies to refine our understanding of intervention parameters.

## Supplementary Information

Supplementary Material 1: Supplementary Table 1: Search Strings used for each database.

Supplementary Material 2: Supplementary Table 2: One-study-removed sensitivity analysis (k = 25, removing each of the 7 pilot studies individually) and pilot study sample sizes by SR-domain.

## Data Availability

The datasets used and analysed during the current study are available from the corresponding author on reasonable request.

## Abbreviations

PA: Physical Activity
SR: Self-Regulation
RoB-2: Risk of Bias 2
MVPA: Moderate-to-Vigorous Physical Activity
HTKS: Head-Toes-Knees-Shoulder
BRIEF-P: Behavior Rating Inventory of Executive Function-Preschool Version
SDQ: Strengths and Difficulties Questionnaire
DCCS: Dimensional Change Card Sort
