# Supplementary material for: The effects of physical activity interventions on preschoolers’ self-regulation skills: a systematic review and meta-analysis of randomized controlled trials
Source: Int J Behav Nutr Phys Act. 2026 Aug 5;23:105. doi: 10.1186/s12966-026-01960-7 (PMC13726147; doi:10.1186/s12966-026-01960-7)
Supplement: Supplementary file 1 [file 12966_2026_1960_MOESM1_ESM.docx]

Search String WEB OF SCIENCE:

((#6 OR #7) NOT ALL=(("Autism" OR "ASD" OR "Autism spectrum disorder" OR "Attention deficit hyperactivity disorder" OR "hyperactivity" OR "ADHD" OR "adolescence" OR "elderly"))) AND (LA==("ENGLISH"))

#6 ("physical activity" OR "exercise" OR "active play" OR "Physical* activ*" OR "Exercis*" OR "play-based activity" OR "movement-based activity") (Title) and ("Self-regulation" OR "self regulation" OR "self- regulat*" OR "self regulat*" OR "behavioral regulation" OR "emotional regulation" OR self-control OR executive-function* OR executive function OR "emotion* regulat*" OR "behavior* regulat*" OR "cognitive regulat*" OR "inhibit* control" OR inhibit* OR switching OR task-switching OR updating OR shifting OR set-shifting OR "cognitive flexibility" OR "working memory" OR "effortful control") (Title) and ("preschool" OR "young children" OR "early childhood" OR child* OR preschool* OR pre-school* OR kindergarten OR daycare OR "day care" OR “childcare” OR “child-care”) (Title)

#7 ("physical activity" OR "exercise" OR "active play" OR "Physical* activ*" OR "Exercis*" OR "play-based activity" OR "movement-based activity") (Abstract) and ("Self-regulation" OR "self regulation" OR "self- regulat*" OR "self regulat*" OR "behavioral regulation" OR "emotional regulation" OR self-control OR executive-function* OR executive function OR "emotion* regulat*" OR "behavior* regulat*" OR "cognitive regulat*" OR "inhibit* control" OR inhibit* OR switching OR task-switching OR updating OR shifting OR set-shifting OR "cognitive flexibility" OR "working memory" OR "effortful control") (Abstract) and ("preschool" OR "young children" OR "early childhood" OR child* OR preschool* OR pre-school* OR kindergarten OR daycare OR "day care" OR “childcare” OR “child-care”) (Abstract)

Search string PSYCINFO:

TI ( ("physical activity" OR "exercise" OR "active play" OR "Physical* activ*" OR "Exercis*" OR "play-based activity" OR "movement-based activity") AND("Self-regulation" OR "self regulation" OR "self- regulat*" OR "self regulat*" OR "behavioral regulation" OR "emotional regulation" OR self-control OR executive-function* OR executive function OR "emotion* regulat*" OR "behavior* regulat*" OR "cognitive regulat*" OR "inhibit* control" OR inhibit* OR switching OR task-switching OR updating OR shifting OR set-shifting OR "cognitive flexibility" OR "working memory" OR "effortful control") AND ("preschool" OR "young children" OR "early childhood" OR child* OR preschool* OR pre-school* OR kindergarten OR daycare OR "day care" OR childcare OR child-care) ) ) OR AB ( ("physical activity" OR "exercise" OR "active play" OR "Physical* activ*" OR "Exercis*" OR "play-based activity" OR "movement-based activity") AND("Self-regulation" OR "self regulation" OR "self- regulat*" OR "self regulat*" OR "behavioral regulation" OR "emotional regulation" OR self-control OR executive-function* OR executive function OR "emotion* regulat*" OR "behavior* regulat*" OR "cognitive regulat*" OR "inhibit* control" OR inhibit* OR switching OR task-switching OR updating OR shifting OR set-shifting OR "cognitive flexibility" OR "working memory" OR "effortful control") AND ("preschool" OR "young children" OR "early childhood" OR child* OR preschool* OR pre-school* OR kindergarten OR daycare OR "day care" OR childcare OR child-care) ) ) OR KW ( ("physical activity" OR "exercise" OR "active play" OR "Physical* activ*" OR "Exercis*" OR "play-based activity" OR "movement-based activity") AND("Self-regulation" OR "self regulation" OR "self- regulat*" OR "self regulat*" OR "behavioral regulation" OR "emotional regulation" OR self-control OR executive-function* OR executive function OR "emotion* regulat*" OR "behavior* regulat*" OR "cognitive regulat*" OR "inhibit* control" OR inhibit* OR switching OR task-switching OR updating OR shifting OR set-shifting OR "cognitive flexibility" OR "working memory" OR "effortful control") AND ("preschool" OR "young children" OR "early childhood" OR child* OR preschool* OR pre-school* OR kindergarten OR daycare OR "day care" OR childcare OR child-care) ) ) NOT ( ("Autism" OR "ASD" OR "Autism spectrum disorder" OR "Attention deficit hyperactivity disorder" OR "hyperactivity" OR "ADHD" OR "adolescence" OR "elderly") )

Search String ERIC:

TI ( ("physical activity" OR "exercise" OR "active play" OR "Physical* activ*" OR "Exercis*" OR "play-based activity" OR "movement-based activity") AND ("Self-regulation" OR "self regulation" OR "self- regulat*" OR "self regulat*" OR "behavioral regulation" OR "emotional regulation" OR self-control OR executive-function* OR executive function OR "emotion* regulat*" OR "behavior* regulat*" OR "cognitive regulat*" OR "inhibit* control" OR inhibit* OR switching OR task-switching OR updating OR shifting OR set-shifting OR "cognitive flexibility" OR "working memory" OR "effortful control") AND ("preschool" OR "young children" OR "early childhood" OR child* OR preschool* OR pre-school* OR kindergarten OR daycare OR "day care" OR childcare OR child-care) ) OR AB ( ("physical activity" OR "exercise" OR "active play" OR "Physical* activ*" OR "Exercis*" OR "play-based activity" OR "movement-based activity") AND ("Self-regulation" OR "self regulation" OR "self- regulat*" OR "self regulat*" OR "behavioral regulation" OR "emotional regulation" OR self-control OR executive-function* OR executive function OR "emotion* regulat*" OR "behavior* regulat*" OR "cognitive regulat*" OR "inhibit* control" OR inhibit* OR switching OR task-switching OR updating OR shifting OR set-shifting OR "cognitive flexibility" OR "working memory" OR "effortful control") AND ("preschool" OR "young children" OR "early childhood" OR child* OR preschool* OR pre-school* OR kindergarten OR daycare OR "day care" OR childcare OR child-care) ) NOT ( ("Autism" OR "ASD" OR "Autism spectrum disorder" OR "Attention deficit hyperactivity disorder" OR "hyperactivity" OR "ADHD" OR "adolescence" OR "elderly") )
