# Supplementary material for: The effects of physical activity interventions on preschoolers’ self-regulation skills: a systematic review and meta-analysis of randomized controlled trials
Source: Int J Behav Nutr Phys Act. 2026 Aug 5;23:105. doi: 10.1186/s12966-026-01960-7 (PMC13726147; doi:10.1186/s12966-026-01960-7)
Supplement: Supplementary file 2 [file 12966_2026_1960_MOESM2_ESM.docx]

**Supplementary Table: One-study-removed sensitivity analysis (k = 25, removing each of the 7 pilot studies individually)**

| **Study removed** | **k** | **Hedges' g** | **95% CI** | **p-value** |
| --- | --- | --- | --- | --- |
| *Kats Gold et al., 2021* | 24 | 0.515 | [0.247, 0.782] | <0.001 |
| *Xiong et al., 2019* | 24 | 0.524 | [0.257, 0.790] | <0.001 |
| *Vazou & Mavilidi, 2021* | 24 | 0.655 | [0.257, 1.053] | 0.001 |
| *Fisher et al., 2011* | 24 | 0.535 | [0.257, 0.793] | <0.001 |
| *Haas et al., 2022* | 24 | 0.535 | [0.268, 0.803] | <0.001 |
| *Pewdee et al., 2024* | 24 | 0.514 | [0.240, 0.787] | <0.001 |
| *Giordano & Alesi, 2022* | 24 | 0.523 | [0.248, 0.798] | <0.001 |

*Note. k = number of studies in the pooled estimate after removal of the indicated study (25 minus 1). Hedges' g range across all removals: 0.514–0.655.*
